# Mining human preference via self-correction causal structure learning

**DOI:** 10.1038/s41598-022-08879-6

**Published:** 2022-03-23

**Authors:** Jian Sun, Chenye Wu, Weihua Peng, Jiayan Huang, Cuiyun Han, Yong Zhu, Yajuan Lyu

**Affiliations:** 1 Institute for Interdisciplinary Information Sciences, Tsinghua University, Beijing, China; 2 School of Science and Engineering, The Chinese University of Hong Kong, Shenzhen, Shenzhen, Guangdong China; 3 Shenzhen Institute of Artificial Intelligence and Robotics for Society, Shenzhen, Guangdong China; 4 Baidu Inc., Shenzhen, Guangdong China; 5 Baidu Inc., Beijing, China

**Keywords:** Human behaviour, Computer science

## Abstract

Spurred by causal structure learning (CSL) ability to reveal the cause–effect connection, significant research efforts have been made to enhance the scalability of CSL algorithms in various artificial intelligence applications. However, less effort has been made regarding the stability and the interpretability of CSL algorithms. Thus, this work proposes a self-correction mechanism that embeds domain knowledge for CSL, improving the stability and accuracy even in low-dimensional but high-noise environments by guaranteeing a meaningful output. The suggested algorithm is challenged against multiple classic and influential CSL algorithms in synthesized and field datasets. Our algorithm achieves a superior accuracy on the synthesized dataset, while on the field dataset, our method interprets the learned causal structure as a human preference for investment, coinciding with domain expert analysis.

## Introduction

Exploring Human Preference (HP, Table 1 lists the main acronyms used in this work) plays an essential role in many areas. Specifically, exploring HP for jobs affects human capital investments^1^; exploring HP for travel service helps improving user profiling for airline industry^2,3^, and accommodation sector^4,5^, and exploring HP for investment advances the financial and capital market, benefiting the whole economy^6^. Generally, HP exploration is a delicate task since it is by nature heterogeneous and can be influenced by each individual’s surrounding environment^7^. The Covid-19 brings a golden opportunity to conduct HP exploration as the global pandemic has drastically changed people’s lifestyles and preferences in this short period^8^. Specifically, this paper focuses on the changes of HP on investment which has attracted much research attention. Most of the literature utilizes statistical methods, such as Friedman Rank Test, Chi-square^6^, and Granger causality^9^. In contrast to the literature, we notice that the changes in HP on investment can be inferred by the different price fluctuation propagation chains between the main financial products before and after the pandemic: different HPs result in different price fluctuation propagation chains, which reveal the cause–effect connections between prices.

**Table 1:** Acronym table.

Acronym	Meaning
Covid-19	Coronavirus disease 2019
HP	Human preference
CSL	Causal structure learning
DAG(s)	Directed acyclic graph(s)
CI(s)	conditional independence(s)
CPDAG(s)	Completed partially directed acyclic graph(s)
DK	Domain knowledge
RCoT	Randomized conditional correlation test
(MA)SHD	(Minimal average) structural hamming distance
MAKL-d	Minimal average Kullback–Leibler divergence
pc.stable	Peter Spirtes & Clark Glymour algorithm (stable version)
RPC*	Robust pc.stable algorithm
iamb(.fdr)	Incremental Association Markov Blanket (with False Discovery Rate Correction) algorithm
fast/inter.iamb	Fast/Interleaved Incremental Association Markov Blanket algorithm
gs	Grow-Shrink algorithm
mmhc	Max-Min Hill Climbing algorithm
h2pc	Hybrid Hybrid Parents and Children algorithm
hc	Hill Climbing search algorithm
tabu	Tabu search algorithm

**Figure 1:**
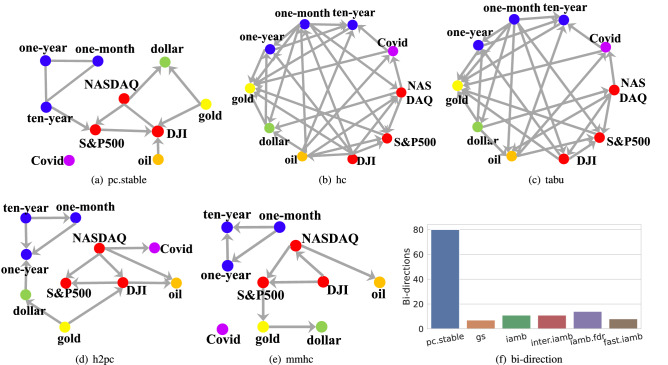
We challenge ten influential CSL algorithms. Six of them are constraint-based: pc.stable^10^, gs^11^, iamb/iamb.fdr^12^, fast.iamb^13^ and inter.iamb^14^. Two are score-based, employing different searching algorithms: hc^15^ and tabu^16^. The remaining two are hybrid algorithms: mmhc^17^ and h2pc^18^. All of them are implemented by employing the bnlearn R package^19^. Five algorithms learn poor structures, which conflict with DK or contain self-conflicts, illustrated in (**a**–**e**). In the structures learned by pc.stable and mmhc, the Covid has no influence on the financial market. Besides, h2pc, tabu and hc suggest financial market can influence the daily confirmed diagnosis. In addition to the real dataset, all constraint-based algorithms are tested on 9 synthetic dataset and the number of self-conflict are counted in (**f**).

Causal Structure Learning (CSL)^20^, one of the mainstream frameworks for causal inference, is a natural candidate for this task because CSL aims at inferring the causal structure presented by Directed Acyclic Graph (DAG), where the directed arrows indicate the cause–effect connections. For example, both raining and opening the sprinkler can make the grass wet, and there is no causal relation between raining (weather condition) and opening the sprinkler (human activities). Suppose a dataset contains three Boolean variables: the status of the rain (Rain), the operation of sprinkler (Sprinkler), and the wetness of the grass (Wet) of each day in a year. CSL could infer a DAG like ‘Rain$\rightarrow$ Wet $\leftarrow$ Sprinkler’.

The classical taxonomy divides CSL into three categories^21^. Constraint-based algorithms^22^ test for conditional independences (CIs) in the data and then find a Markov equivalence class of DAGs (rather than the specific DAG) that best explains these independences since some DAGs are observational equivalent. Each Markov equivalence class can be represented by a Completed Partially Directed Acyclic Graph (CPDAG)^23^ that involves both directed and undirected edges shared among all class members. Score-based algorithms^24^ define a hypothesis space of possible DAGs and a scoring function that measures how well the DAG fits the observed data, and then such algorithms find the highest-scoring DAG. Thus, they do not rely on hypothesis testing as much as constraint-based ones. However, different hypothesis space and scoring functions can result in different “best” DAGs even on the same dataset. Hybrid algorithms^17^ combine the above two schemes by learning the graph skeleton through a constraint-based algorithm, using CIs as constraints to construct the skeleton, and then employing the score-based algorithm for evaluating causal graphs through a goodness-of-fit score function. Comprehensive comparison analysis is available^25^ for interested audience. Among all methods, the constraint-based ones are the most intuitive and most processing efficient^22^.

However, CSL, even using constraint-based algorithms, can produce meaningless structures. Take the impact of Covid-19 as an example, a widely adopted domain knowledge (DK) is that the rapid spread of Covid-19 has dramatic impacts on financial markets all over the world^26^. Thus, in the causal structure between the price of main financial products after the outbreak of Covid-19 and the daily confirmed diagnosis of U.S. data, there should be arrows pointing at the financial products from Covid-19. However, Fig. 1a–e show that many learned causal structures not only fail to reflect the DK but even contradict the DK.

In addition to conflicting with DK, CSL results can also contain self-conflict. We again take constraint-based algorithms to exemplify this phenomenon better. One type of self-conflict is an edge oriented into two opposite directions during the algorithms, termed as bi-oriented edges. Figure 1f counts the number of these bi-oriented edges of 6 constraint-based algorithms on nine synthetic datasets. The self-conflict significantly reduces the credibility of the whole algorithm since constraint-based algorithms cannot contain the error propagation, as shown by the blue part in Fig. 3 (The details will be introduced later.). Such errors could mislead the entire procedure^21^ due to the lack of a self-correction mechanism.

**Figure 2:**
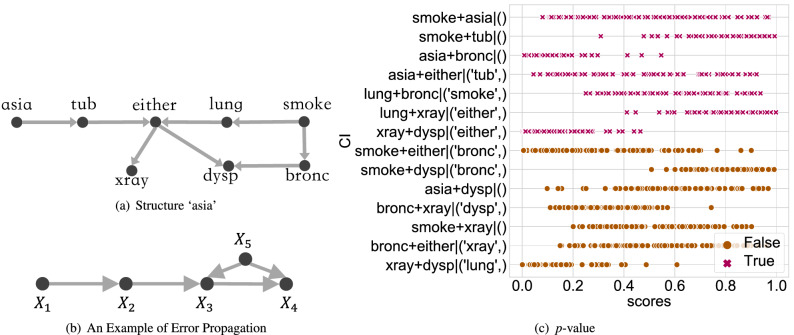
Traditional constraint-based algorithm analysis. We take the state-of-the-art algorithm, Randomized conditional Correlation Test (RCoT)^28^, as an example. The tested dataset is generated from the structure shown in (**a**). The generation process is similar to that elaborated in *Synthetic Data* part. Several CI hypotheses are selected, and RCoT is conducted 100 times on each CI hypothesis, with the returned *p* values shown in (**c**). The *y*-axis shows CI hypotheses in the form of $X_i+X_j|{\mathbb {Z}}$. The *x*-axis shows the corresponding 100 *p* values. The red forks are the *p* values of the CI hypotheses that should be accepted, and the brown dots should be rejected. However, it is hard to statistically distinguish whether a CI hypothesis should be rejected or not, making the decisions solely based on CI testings not adequately convincing. (**b**) shows an example of CI testing error propagating, which may ultimately mislead the entire algorithm.

**Figure 3:**
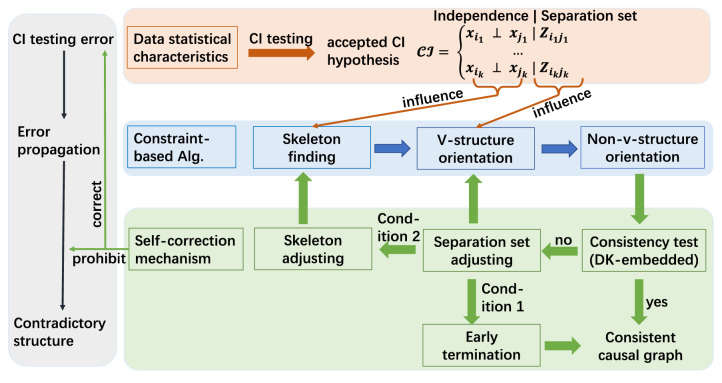
The gray part broadly highlights why constraint-based algorithm can produce contradictory results. The orange part shows how the CI testing results influence each step of constraint-based algorithms. The blue part broadly introduces the processes of constraint-based algorithms. The green part is how we build a self-correction mechanism to revise the CI testing results and get consistent causal structures.

We submit that these conflicts, including conflict with DK and self-conflict, come from two aspects, as shown by the gray part in Fig. 3. The first source is that constraint-based algorithms are highly dependent on CI testing. On the one hand, this dependence contributes to the clear intuition of constraint-based algorithms; on the other hand, the CI testing accuracy can fundamentally affect the performance of the entire algorithm. However, in practice, the *p* value returned by the CI testing (the probability of a given CI hypothesis being true), is not reliable, as essentially, testing whether two random variables are independent (especially continuous variables) is a complex statistical problem^27^. Although many researchers aim to develop accurate CI testing algorithms, state-of-the-art algorithms are not flawless. Many true CI hypotheses still cannot be distinguished from the false ones, especially in the high-noise environment, as shown in Fig. 2. Thus, the decisions solely based on CI testings are not adequately convincing. The second source involves the CI testing error propagating, which may ultimately mislead the entire algorithm, with^22^ providing a relevant example. Let the ground truth be illustrated in Fig. 2b. If the edge $X_5-X_4$ is mistakenly removed, then the edge $X_2-X_4$ will not be removed because $X_2$ and $X_4$ are dependent on every subset of their neighbor variables $\{X_1,X_3\}$. One may argue that the propagation will not exist if the independence is tested conditioning on every subset of all other variables $\{X_1,X_3, X_5\}$. This approach suffers from high computational complexity. Moreover, although the error is no longer propagated, it cannot be corrected.

To summarize, CI testing errors are unavoidable, and they easily propagate since it is the only approach to characterizing the accepted CI hypothesis. In order to overcome this issue, inspired by the fact that a wrongly accepted/rejected CI hypothesis may cause conflicts during the edge orientation, this work suggests exploiting the consistency of causal structures. For example, when some accepted CI hypotheses suggest that the edge $X_1-X_2$ should be oriented as $X_1\rightarrow X_2$, while others indicate the opposite, this orientation difference indicates that some CI hypotheses have been wrongly accepted/rejected. Spurred by this idea, we design a CSL algorithm with a DK-embedded self-correction mechanism (termed as RPC* algorithm), which is proven to be complete, to correct CI testing errors and prohibit the error propagation, as shown by the green part of Fig. 3.

The DK generated from experience, knowledge graph, or other sources contributes to our proposed solution from two aspects. First, it accelerates the process by directly pointing out the wrongly accepted/rejected CIs, and second, it checks whether the DK criterion is satisfied as part of the consistency test. Thus, the output of the proposed algorithm is always consistent with DK. Besides, the self-correction mechanism enhances our algorithm’s stability and accuracy even in a high-noise environment where CI testing errors are more likely to exist. Hence, the output is guaranteed to be consistent and meaningful by combining the above two processes. The proposed algorithm can be effective in mining various HPs, much beyond HP for investment. Table 2 positions our proposed algorithm (RPC*) in the literature.

**Table 2:** Position of this work in the literature.

	pc.stable	gs	iamb(.fdr)	fast/inter.iamb	hc/tabu	h2pc/mmhc	RPC*
Fully oriented graph	$\times$	$\times$	$\times$	$\times$	$\checkmark$	$\times$	$\checkmark$
DK embedded	$\times$	$\times$	$\times$	$\times$	$\checkmark$	$\checkmark$	$\checkmark$
Constraint embedded	$\checkmark$	$\checkmark$	$\checkmark$	$\checkmark$	$\times$	$\checkmark$	$\checkmark$
Self-correction mechanism	$\times$	$\times$	$\times$	$\times$	$\times$	$\times$	$\checkmark$

## Results

We employ two types of datasets. One type is the synthetic dataset, where the ground truth structure, $G_t$, is known since the dataset is generated according to the structure. The other type is the real-life dataset, where the ground truth is unknown. For the first dataset type, the evaluation metric measures the distance between the learned structure and the ground truth structure. The traditional distance metric is the Structural Hamming Distance (SHD)^17^ in which any difference between variable pairs counts. Generally, SHD increases with the number of variables (data dimension), i.e., $|{\mathbb {V}}|$. Due to the diversity of the employed datasets, we adopt the ratio between the SHD and $|{\mathbb {V}}|$ to unify the performance metrics among the various datasets. The minimal ratio that an algorithm can achieve (termed as MASHD), measures the algorithm’s accuracy. The lower the MASHD, the better the algorithm performs.

Unfortunately, MASHD cannot be employed for the second type of dataset since $G_t$ is unknown. For this type of dataset, one popular measurement is KL-divergence^17^, with the KL-divergence of a DAG *G* measuring the distance between the dataset and the distribution generated by *G*. Since the CSL algorithms’ outputs are CPDAGs, i.e., [${\hat{G}}$], we first enumerate all possible orientations of the undirected edges in CPDAGs, i.e., all $G\in {\hat{G}}$, and select the final output as the one with the maximal ratio between the KL-divergence and $|{\mathbb {V}}|$ (MAKL-d). The higher the MAKL-d, the better the algorithm performs.

We challenge the proposed method against ten influential CSL algorithms. Six of them are constraint-based: pc.stable^10^, gs^11^, iamb/iamb.fdr^12^, fast.iamb^13^ and inter.iamb^14^ and two are score-based, employing different searching algorithms: hc^15^ and tabu^16^. The remaining two are hybrid algorithms: mmhc^17^ and h2pc^18^. All methods are implemented employing the bnlearn R package^19^. Although the hybrid and our proposed RPC* algorithm exploit DK, they process DK quite differently, as the hybrid algorithms utilize DK to generate a goodness-of-fit score function for evaluating causal graphs while DK in RPC* helps to check the correctness of CIs and the consistency of causal graphs.

### Synthesized data analysis

**Table 3:** Synthesized dataset generation.

(a) Structures						(b) Six adopted noise types			
Name	Cancer	Survey	Asia	Sachs	Child	Type\$$\sigma (\eta _i)$$	0.5	1	2
Node	5	6	8	11	20	Gaussian	N(0,0.25)	N(0,1)	N(0,4)
Edges	4	6	8	17	25	Uniform	U($$-\frac{\sqrt{3}}{2}$$, $$\frac{\sqrt{3}}{2}$$)	U($$-\sqrt{3}$$, $$\sqrt{3}$$)	U($$-2\sqrt{3}$$, 2$$\sqrt{3}$$)

**Figure 4:**
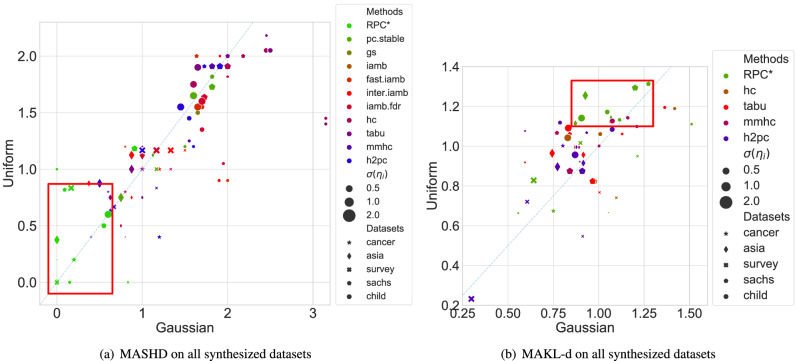
Performance on synthesized datasets.

Utilizing Bayesian Network Repository^29^, we employ five structures of different dimensions as benchmarks with their detailed information presented in Table 3a. For each structure, the datasets are generated based on the following mechanism:

1 $$\begin{aligned} X_i = \sum \limits _{j\in \mathbf{P} {} \mathbf{a} _i} f_j(X_j) + \eta _i, \end{aligned}$$

where $f_j(\cdot )$ is a function that is randomly selected from $\{sin(\cdot ), cos(\cdot ), arctan(\cdot ), abs(\cdot ), square(\cdot )\}$, as these provide adequate complexity (without being linear) and their domains are ${\mathbb {R}}$. $\eta _i$ denotes noise. We apply six noise distributions involving different noise types and different noise standard deviation $\sigma (\eta _i)$ (see Table 3b). Each distribution is related to one dataset, i.e., each structure generates six datasets. In each dataset, the noise distribution obeys one of the distributions in Table 3b.

We present how each algorithm performs in terms of MASHD for all datasets in Fig. 4a. The *x*-axis shows the performance on the dataset affected by Gaussian noise, and the *y*-axis shows the performance under Uniform noise. Different shapes of markers distinguish different structures, while the size of markers presents the standard deviation of the noise. We conclude that in terms of MASHD, our algorithm outperforms all competitors in all datasets. Additionally, one general trend is that when the standard deviation of the noise is the same, the Uniform noise seems to degrade the performance slighter than the Gaussian noise since most markers lie under the dashed diagonal line. However, as the noise standard deviation increases, although the performance of the challenged algorithms is relatively stable for the Gaussian noise, the performance degrades significantly for Uniform noise since big markers mainly lie above the diagonal line. In contrast, small markers mainly lie under the line. It should be noted that the suggested RPC* method manages a stable performance regardless of the noise type.

Figure 4b presents the MAKL-d performance on all datasets where only four challenged algorithms generate meaningful structures. The RPC* algorithm still outperforms the competitors on most datasets, whether in terms of MAKL-d or stability since the performance of many algorithms degrades as the noise standard variance increases. This highlights the superior of RPC* among different performance metrics.

Additionally, as there is no DK for the synthesized dataset, the DK is not embedded in the algorithms. However, even so, RPC* still shows superior performance against challenged algorithms, which confirms the ability of RPC* to find true causal structures. For more detailed comparisons results on the synthetic datasets, the detailed results of Fig. 4a,b are provided in “Method”.

### Real case analysis

To analyze how Covid-19 changes the investor’s preference, we explore the causal structure among the prices of main financial products before and after the outbreak of Covid-19. We select five main financial products and collect the related data^30^:

1. *Stocks*: DJI (Dow Jones Industrial Average), S&P500 (Standard and Poor’s Composite 500 Index), NASDAQ (National Association of Securities Dealers Automated Quotations);
2. *U.S. Treasury Securities*: one-month/one-year/ten-year treasury bond yield;
3. *Gold*: the price of the gold CFD (Contract For Difference) in Intercontinental Exchange;
4. *Oil*: the price of WTI (West Texas Intermediate) crude oil futures;
5. *Dollar*: the U.S. dollar index;

The chosen products are commonly used when analyzing the dynamics of financial markets^31–35^. All the above data were the closing prices collected before the Covid-19 outbreak, i.e., from January 2, 2019, to January 21, 2020, and after the outbreak, from January 22, 2020, to December 15, 2020. The specific dates are chosen based on the following two reasons. January 22, 2020, was the dividing line indicating the outbreak of Covid-19 since it is the earliest record of the daily confirmed diagnosis of the U.S. data repository by the Johns Hopkins University^36^. On the other hand, to avoid a possible impact of imbalanced datasets, their sizes before and after the break are roughly the same. In the raw data, these variables lie naturally in different ranges. For example, the observations of DJI are in the thousands while the treasury bond yields are percentages. Thus to present the trend uniformly, for each variable $\mathbf{x}$, its observations are scaled into range [0, 1]:

2 $$\begin{aligned} x_i^{scaled} = \frac{x_i - min\{\mathbf{x}\}}{max\{\mathbf{x}\}-min\{\mathbf{x}\}}, \forall i. \end{aligned}$$

**Figure 5:**
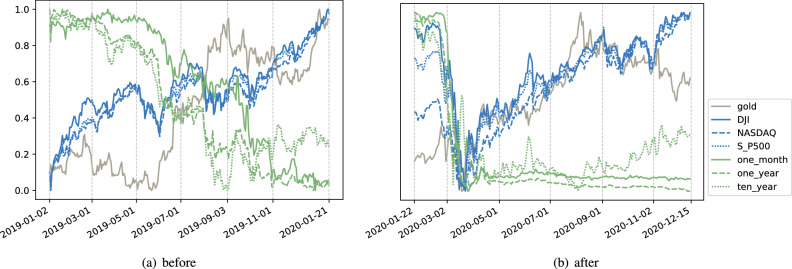
Changes on stock, securities and gold before and after the outbreak of Covid-19.

The scaled stock index, treasury bond yield, and gold price are plotted in Fig. 5a,b. Figure 5a reveals the treasury bond yield and the gold price often act negatively to the stock index while shortly after the Covid-19 outbreak (Fig. 5b), although the treasury bond still presented a negative reaction, the gold price reacted positively. Although this superficial observation may not directly reveal how the investment preference changes, it strongly suggests different preferences before and after the Covid-19.

Concerning the Covid-19 example, when the algorithm generates the DAGs by exploiting data after the Covid-19 outbreak, except checking the DAGs’ consistency, the DK should also be ensured in terms of financial products not influencing the daily confirmed diagnosis results. Thus, any DAG containing an arrow pointing at “Covid” is meaningless and will not be considered. Additionally, if an algorithm does not generate a meaningful DAG, it cannot be named flawless.

**Figure 6:**
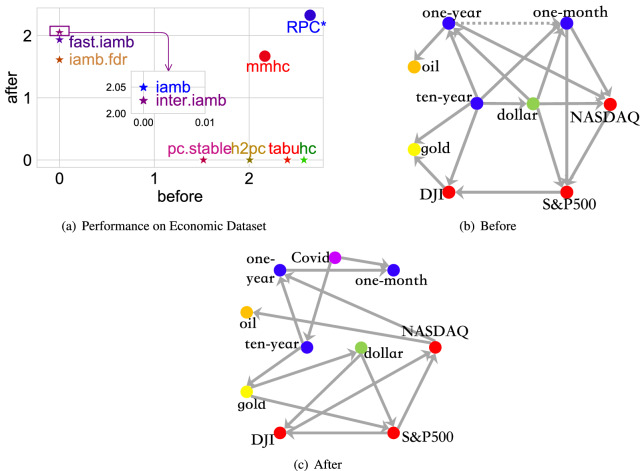
Influence of Covid-19 on causal structures between financial products.

Given that the true causal structure of these financial products is unknown, the only quantitative metric to evaluate the DAGs is MAKL-d, with the corresponding results presented in Fig. 6a. The *x*-axis shows the MAKL-d on the dataset before the Covid-19 outbreak, while the *y*-axis indicates after the outbreak. If an algorithm cannot generate meaningful structures on one of the two periods, the related MAKL-d is recorded as 0, and the algorithm will be associated with a star. Algorithms that cannot generate meaningful structures for both periods are omitted. Figure 6a highlights that only two algorithms, i.e., the proposed algorithm and mmhc (presented by dots), generate meaningful structures on both periods (presented by dots), and the proposed method shows the superiority against mmhc.

Figure 6b,c compare the structures affording the highest MAKL-d before and after the outbreak of Covid-19, with different products being plotted in different colors. Although more than one DAG may share the highest MAKL-d, for example, before the Covid-19 outbreak, two DAGs have the highest MAKL-d, in these two DAGs, only one edge is oriented in opposite directions, i.e., the edge between one year and one month, plotted as dashed in Fig. 6b.

The causal structure with the highest MAKL-d has the highest probability of revealing the price fluctuation propagation chain between the main financial products, i.e., the HP for investment. Before the outbreak, the only source is the Ten-year treasury bond, indicating its high priority during investment^37^. Note that, such priority does not mean the yield will decrease or increase. It merely infers that the price fluctuation of this product is the cause of price fluctuations of other products, thus reflecting this product is considered for investment before investing in other products. Besides the Ten-year treasury bond, the dollar is of the second-highest priority. Both facts suggest the strong confidence of investors in the U.S. economy^38^. Then the price fluctuation propagation chain continues with the short-term treasury bond, oil/stock. This coincides with the fact that the value change of the dollar will affect the stock indexes since dollars are needed to purchase stocks^39^. Finally, gold acts as the safe haven since the value of gold lies in preservation rather than investment^35,40^. Overall, this chain (i.e., the HP for investment) shows a positive attitude of investors toward the economy.

However, the pandemic changes this investment attitude and the price fluctuation propagation chain. After the outbreak of Covid-19, the directly affected products are the treasury bonds, which is consistent with the statement that the Covid-19 has increased the persistence degree of bonds^41^. No matter before or after the outbreak, treasury bonds are the source of the price fluctuation propagation chain, supporting their role as an anchor for global asset prices^42^. Then the yield fluctuation is passed to the price of gold rather than dollar/stock. In other words, after the outbreak, investments consider investing in gold before investing in dollar/stock, which is the opposite of before the outbreak. The same circumstance also happened in the euro-area where the flight to safety phenomenon^43^ moved financial agents away from the more risky assets and towards the safer investment-grade segment^44^. This observation shows the significant change in the investment subconscious due to the negative attitude of investors towards the economy. This subconscious change is also supported by a poll released by Gallup Wednesday, which shows 67% of Americans believe economic conditions are getting worse in the country^45^. Similarly, the same statement was delivered by the researchers of the Reserve Bank of India^46^. The alignment between the interpretation of the causal structure and expert experience strongly suggests the effectiveness of RPC*.

Considering distinguishing the effect of the regulatory directives from HP shifts, the analysis comes from two aspects. For the long-term regulatory, such as new legislation, if data is collected in the short time following the emergency that results in the HP shift, as in our case, it should not affect the learned causal structure. For the quick-acting short-term regulatory directives, such as the circuit breaker, on the one hand, they are often pre-known, and thus their effects are always foreseeable when the regulation directives are triggered. However, the extreme market condition is a reflection of sudden HP shifts, which is not foreseeable due to the limited knowledge of the emergency. Therefore, although the regulation can affect the market, it can be considered as the effects of sudden HP shift on the market. On the other hand, the short-term regulatory may affect market conditions for only a short period, and hence cannot influence the statistical relation between market variables.

## Discussion

This work focuses on adopting CSL for exploring HP in a high-noise environment. In such an environment, traditional algorithms fail as they rely on hypothesis testing, of which the statistical errors make the outcome lose stability and explainability. Spurred by that, we creatively propose a self-correction mechanism combined with a consistency test to correct the wrong hypothesis testing result and guarantee the explainability of the learned structure even in a high-noise environment. We challenge our method against several state-of-the-art algorithms on various datasets and demonstrate the superiority of our algorithm on both synthetic and real data.

This work can be extended in many directions. For example, employing the adjacency matrix for batch operation may relieve the computational issue in our algorithm. In addition, our algorithm has many important application domains, such as user profile, pattern recognition, and policy evaluation.

This work is inherited from the constraint-based algorithm, where the basic assumption is that all concerned variables are observational, i.e., there is no latent structure. Specifically, latent structure analysis is another mainstream research in CSL^47,48^. However, since the latent structure is not in the scope of this work, there is no guarantee that the proposed algorithm is complete when the latent variables exist. Besides, scalability is one common concern for constraint-based algorithms. We emphasize that there are many important areas, such as biology, and financial markets, where the number of concerned variables is relatively small, and it is worthwhile to figure out the consistent causal structure regardless of the computational efforts.

## Methods

### Algorithm framework

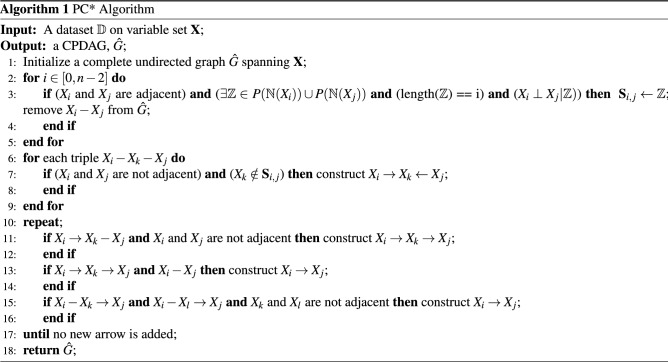



Many constraint-based algorithms like Grow-Shrink^49^ and Interleaved Incremental Association^50^ are inherited from the Peter and Clark (PC*) algorithm^22^, which contains three main steps, as shown by the blue part in Fig. 3, *Skeleton finding*, *V-structure orientation* and *Non-v-structure orientation*. These steps are affected by different components of accepted CI hypotheses separately, as the orange part in Fig. 3 shows. Specifically,

- *Skeleton finding*: The skeleton of a DAG is the undirected graph obtained by replacing all directed arrows with undirected edges. Take the DAG ‘Rain$\rightarrow$ Wet $\leftarrow$ Sprinkler’ as an example; the skeleton is ‘Rain-Wet-Sprinkler’. Thus, the *Skeleton finding* determines whether there are edges between variable pairs based on the independence of the accepted CI hypothesis. The procedure *Skeleton finding* is shown in lines 2-5 in Algorithm 1.
- *V-structure orientation*: A v-structure is a sub-structure of a DAG comprising three variables $X_i\rightarrow X_j \leftarrow X_k$ where $X_i$ and $X_k$ are not adjacent. ‘Rain$\rightarrow$ Wet $\leftarrow$ Sprinkler’ is a v-structure. The *V-structure orientation* determines the v-structures based on the separation set. The procedure *V-structure orientation* is illustrated in lines 6-9
- *Non-v-structure orientation*: Non-v-structures are oriented based on the rules (lines 11-16) which are proven to make the output complete^20^. The procedure *Non-v-structure orientation* is indicated in lines 10-18.

Note that, in "Skeleton finding", only the independence matters. For example, let ${\mathbb {Z}}$ be the actual separation set for a variable pair $(X_i, X_j)$, but the CI hypothesis $X_i\perp X_j|{\mathbb {Z}}'$ is accepted. Then edge $(X_i, X_j)$ is deleted, and ${\mathbb {Z}}'$ is recorded as the separation set for $(X_i, X_j)$. In this case, the separation set is incorrect, but the output skeleton can still be correct.

Based on the above analysis, we propose an algorithm termed as Robust PC* (RPC*), which is illustrated by the blue part and the green part of Fig. 3. RPC* starts with generating an accepted CI hypothesis set, ${{\mathbb {C}}}{{\mathbb {I}}}$ in "Skeleton finding". Then the two processes, “Skeleton adjusting” and "Separation set adjusting" are used to adjust the independence and the separation sets in ${{\mathbb {C}}}{{\mathbb {I}}}$ respectively. Generally, if ${{\mathbb {C}}}{{\mathbb {I}}}$ is consistent, the algorithm outputs consistent CPDAGs. Otherwise, assuming the skeleton is right, i.e., the independence is correct, the separation sets are adjusted until consistent CPDAGs are found. The skeleton is adjusted if no consistent CPDAGs can be found (Condition 2). This process terminates if the iterations reach a threshold (Condition 1). Specifically, RPC* considers all possible causes for inconsistent structures and embeds the corresponding adjustments. Thus, RPC* is complete. The detailed algorithm design is shown in the next subsections.

### Self-correction CSL

#### Skeleton finding

As stated earlier, it is risky to determine whether to accept a CI hypothesis solely relying on a single *p* value. Hence, to improve the resistance against noise, we propose a compound score that measures the probability of a CI hypothesis to be true. Given a CI hypothesis $X_i\perp X_j|{\mathbb {Z}}$ and $p_t$ denoting the $t^{th}$ calculation of *p* value, the score of the CI hypothesis $S(X_i, X_j|{\mathbb {Z}})$ is

3 $$\begin{aligned} S(X_i, X_j|{\mathbb {Z}}) {:}{=}\sum \limits _{t=1}^T I(p_t > \alpha ) + \frac{\sum _{t=1}^T p_t}{T}, \end{aligned}$$

where $I(\cdot )$ is the indicator function, *T* is the total number of *p* value calculations, and $\alpha$ is a predefined threshold. Setting the value of *T* is a trade-off between stability and efficiency. The traditional way that employs a single *p* value is a special case of $S(X_i, X_j|{\mathbb {Z}})$ with $T = 1$ and $\alpha > 1$.

Nevertheless, to characterize an initial ${{\mathbb {C}}}{{\mathbb {I}}}$, an additional predefined threshold $\beta$ is required, which accepts the hypothesis and adds it into ${{\mathbb {C}}}{{\mathbb {I}}}$ when the score is above $\beta$. It should be noted that accurately setting $\beta$ is unnecessary, as the process to correct the ${{\mathbb {C}}}{{\mathbb {I}}}$ is elaborated in “Separation set adjusting”.

#### Consistency test

**Figure 7:**
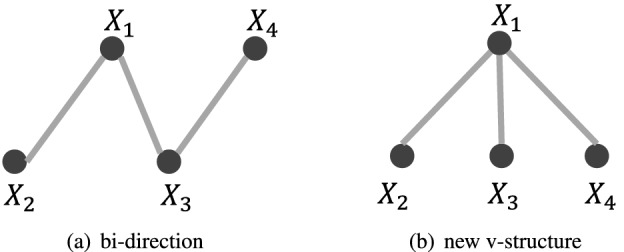
Cases violating the consistency conditions. Suppose that the accepted CI hypotheses set is $\{X_1\perp X_4, X_2 \perp X_3, X_2\perp X_4| \{X_1, X_3\}\}$. The introduced skeleton is shown in (**a**), where $X_1\rightarrow X_3\leftarrow X_4$ forms a v-structure based on the first CI hypothesis, and $X_2\rightarrow X_1\leftarrow X_3$ forms another v-structure based on the second CI hypothesis. Edge $X_1-X_3$ is oriented in the opposite directions. (**b**) A new v-structure, where ${{\mathbb {C}}}{{\mathbb {I}}}$ is $\{X_3\perp X_4, X_2 \perp X_3, X_2\perp X_4| \{X_1, X_3\}\}$. In its skeleton, $X_2 \rightarrow X_1\leftarrow X_3$ and $X_3\rightarrow X_1\leftarrow X_4$ form two v-structures. Thus, there should be an additional v-structure $X_2\rightarrow X_1\leftarrow X_4$. However, this v-structure cannot be produced in the *v-structure orientation*.

**Figure 8:**
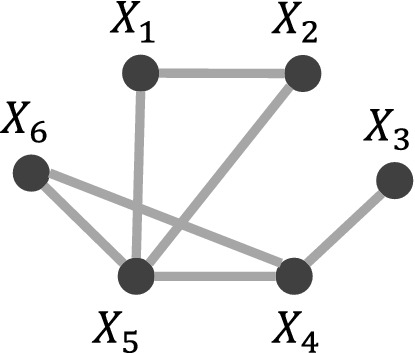
Early termination: considering this figure as an example, in the *V-structure orientation* process, the CI hypothesis $X_3\perp X_5|X_6$ will form a v-structure $X_5\rightarrow X_4\leftarrow X_3$. Thus we record $(X_4, X_5)$ for $X_5\rightarrow X_4$ and $(X_3, X_4)$ for $X_3\rightarrow X_4$. Following the same idea, we record $(X_5, X_6)$ for $X_6\rightarrow X_5$ since $X_2\perp X_6|X_4$. Then the *rules* process orients $X_4\rightarrow X_6$, otherwise a new v-structure $X_3\rightarrow X_4\leftarrow X_6$ will be produced. Then we record $(X_3, X_4)$ for $X_4\rightarrow X_6$ since the orientation of $(X_3, X_4)$ is the reason to orient $X_4\rightarrow X_6$. Now a cycle $X_6\rightarrow X_5 \rightarrow X_4 \rightarrow X_6$ is detected. $(X_4, X_5), (X_4, X_5), (X_5, X_6)$ are reported since they are the evidence path of the cycle’s edges. The most recorded pair is $(X_4, X_5)$, and the related CI hypotheses are $X_2\perp X_4|\{X_1, X_6\}$ and $X_3\perp X_5|X_6$. These will not form v-structures by decorating the former one as $X_2\perp X_4|\{X_1, X_5, X_6\}$ and the latter one as $X_3\perp X_5|\{X_4, X_6\}$.

A structure ${\hat{G}}$ is said to be consistent *if and only if (iff)* ${\hat{G}}$ satisfies a) no edge will be oriented in opposite directions, b) ${\hat{G}}$ does not contain any directed cycle, c) all v-structures in ${\hat{G}}$ are oriented in *V-structure orientation* and *Non-v-structures orientation* will not introduce new v-structures, and d) ${\hat{G}}$ does not conflict with DK. Set ${{\mathbb {C}}}{{\mathbb {I}}}$ is consistent *iff* it introduces a consistent CPDAG. Figures 7a,b and 8 present the cases violating the consistency condition (a), (b) and (c), respectively. It is worth noting that the consistency test is not redundant since the inconsistency occurs (as addressed in Fig. 1f) due to the wrongly accepted/rejected CI hypotheses. A common deficiency of several constraint-based algorithms is that they do not precisely evaluate consistency. Instead, they randomly choose one direction when the first condition is violated.

#### Separation set adjusting

In this process, we assume that ${{\mathbb {C}}}{{\mathbb {I}}}$ introduces the correct skeleton $({\mathbb {V}}, {\mathbb {E}})$ while the separation sets of some accepted CI hypotheses are wrong. Specifically, we first introduce the separation sets that should be adjusted and how to adjust them. A detailed explanation is given in Algorithm 2 at the end of this subsection.

A simplistic approach to find the correct separation set for each accepted CI hypothesis is to test all possible separation sets and select the one with the highest score. However, this strategy is rather time-consuming, involving many unnecessary CI tests, as during the *V-structure orientation* process, not all accepted CI hypotheses are essential for determining the directions. For example, the skeleton of Fig. 2b highlights that regardless of the exact separation set of $(X_1, X_5)$, it does not affect the procedure *V-structure orientation* since $X_1, X_5$ cannot constitute any v-structure. What matters are the v-structures. Hence, the non-connected variable pairs with common neighbors require greater attention since these pairs can construct v-structures. Therefore, all possible separation sets should be considered for these variable pairs. Mathematically, the following CI hypotheses should be tested:

4 $$\begin{aligned} X_i\!\perp \! X_j | {\mathbb {Z}}, X_i-X_j\notin {\mathbb {E}}, {\mathbb {N}}(X_i)\!\cap \! {\mathbb {N}}(X_j)\! \ne \! \varnothing , {\mathbb {Z}}\! \in \! P({\mathbb {N}}(X_i))\!\cup \! P({\mathbb {N}}(X_j)), \end{aligned}$$

where ${\mathbb {N}}(\cdot )$ is the neighbor variables set and $P(\cdot )$ is the power set.

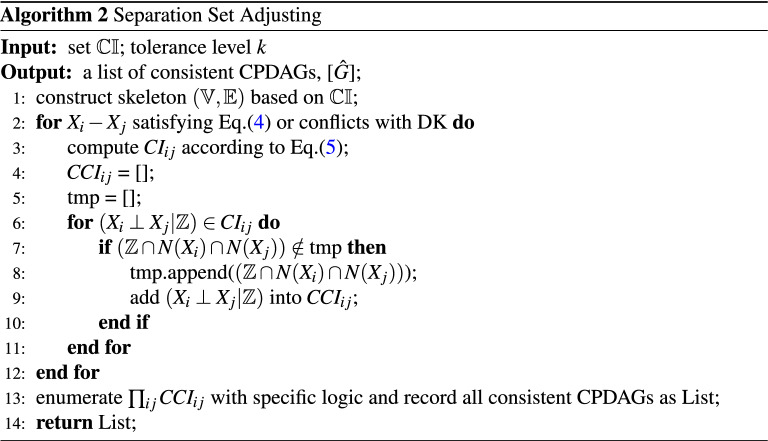



Additionally, if any CI hypothesis conflicts with DK, the related variable pairs also require greater attention. For example, one DK is that generally, the spread of the virus will not be directly affected by the stock market. Thus, if the accepted CI hypothesis leads to a directed edge “stock price $\rightarrow$ daily confirmed diagnosis of Covid-19”, it will be considered a false CI hypothesis. All other possible separation sets should be tested.

After testing all possible CI hypotheses of these suspicious variable pairs, the algorithm adjusts their separation sets. *k* CI hypotheses with the highest scores are considered for each suspicious variable pair to attain robustness to noise. These CI hypotheses are denoted as:

5 $$\begin{aligned} CI_{ij}{:}{=}\{X_i\!\perp \! X_j|{\mathbb {Z}}, rank(S(X_i,X_j|{\mathbb {Z}}))\!\le \! k\}, \end{aligned}$$

where $rank(\cdot )$ refers to the score ranking of all CI hypotheses tested and parameter *k* is a predefined threshold. All tested separation sets for $X_i-X_j$ are recorded into a set ${{\mathbb {T}}}{{\mathbb {S}}}_{ij}$. Notice that some separation sets are just identical for *V-structure orientation*. Considering the skeleton of Fig. 2b as an example, both CI hypotheses $X_2\perp X_5|\{X_3\}$ and $X_2 \perp X_5 | \{X_1, X_3\}$ do not result in any v-structure, while both $X_2\perp X_5$ and $X_2 \perp X_5 | \{X_1\}$ produce the same v-structure $X_2 \rightarrow X_3 \leftarrow X_5$. Thus, only a subset of $CI_{ij}$ denoted as $CCI_{ij}$ (compact $CI_{ij}$) must be enumerated since its members produce different v-structures. The details are shown in line 4 to line 11 in Algorithm 2.

The next step is for each $X_i-X_j \notin {\mathbb {E}}$, choosing a CI hypothesis from $CCI_{ij}$ to construct a consistent CI hypothesis set ${{\mathbb {C}}}{{\mathbb {I}}}'$. If all candidate CI hypothesis sets are enumerated, the algorithm stops when the first consistent CPDAG is delivered, and the output contains only one CPDAG. However, the output variety affords robustness to higher noise levels. To guarantee the output variety, one method is to iteratively replace *L* CI hypotheses with the lowest scores in ${{\mathbb {C}}}{{\mathbb {I}}}$ and record all consistent CPDAGs where *L* is an integer increasing from 1.

*Remark*: Although in "Skeleton finding", the initial CI hypothesis set ${{\mathbb {C}}}{{\mathbb {I}}}$ is generated by a predefined parameter $\beta$, the strategy seems inflexible. The inflexibility vanishes in *Separation set adjusting*. There is a chance that no fixed threshold can produce the final accepted CI hypothesis since the CI hypothesis with a lower score can be accepted while the one with a higher score can be rejected. For example, even if $S(X_i, X_j|{\mathbb {Z}}) > S(X_i, X_j |{\mathbb {Z}}')$, it is still possible that only $X_i \perp X_j | {\mathbb {Z}}'$ is accepted since it contributes to making up a consistent CI hypothesis set while the other does not.

#### Skeleton adjusting

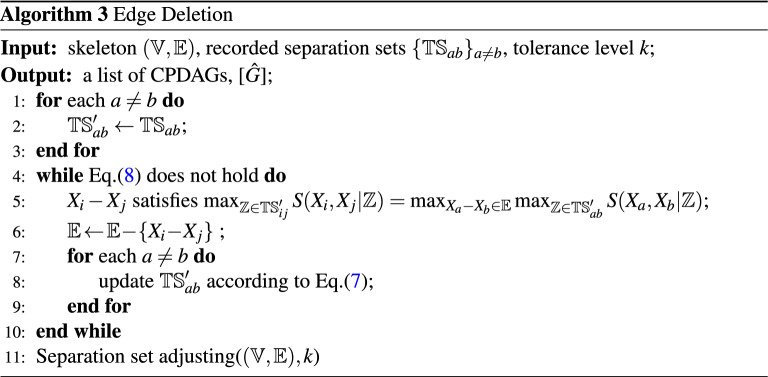

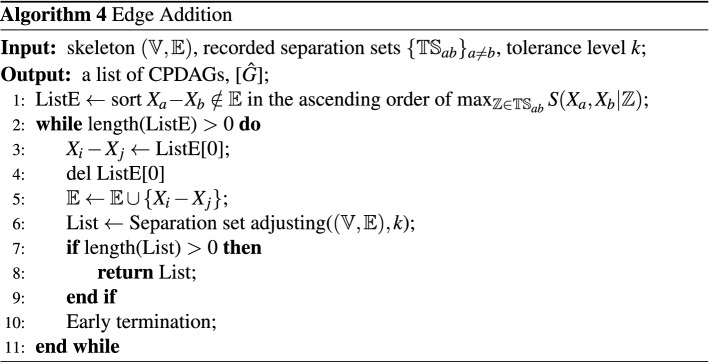





If no consistent CI hypothesis set can be found in the above process, i.e., condition 2 in Fig. 3, the remaining possibility is that the skeleton $({\mathbb {V}}, {\mathbb {E}})$, introduced by ${{\mathbb {C}}}{{\mathbb {I}}}$ is wrong. For each wrongly processed edge, the edge can be mistakenly deleted or preserved. For the mistakenly preserved case, suppose $X_i-X_j$ is the edge that has the highest score in ${\mathbb {E}}$. Mathematically, if

6 $$\begin{aligned} \max _{{\mathbb {Z}} \in {{\mathbb {T}}}{{\mathbb {S}}}_{ij}}S(X_i, X_j|{\mathbb {Z}}) = \max _{X_a-X_b\in {\mathbb {E}}}\max _{{\mathbb {Z}} \in {{\mathbb {T}}}{{\mathbb {S}}}_{ab}}S(X_a, X_b|{\mathbb {Z}}), \end{aligned}$$

then $X_i-X_j$ has the highest probability to be wrongly preserved. If edge $X_i-X_j$ is deleted, the scores of CI hypotheses originating from other variable pairs will change since $X_i$ and $X_j$ are no longer neighbors, and thus ${{\mathbb {T}}}{{\mathbb {S}}}_{ab}$ should be updated accordingly:

7 $$\begin{aligned} {{\mathbb {T}}}{{\mathbb {S}}}'_{ab} {:}{=}\{{\mathbb {Z}}\in {{\mathbb {T}}}{{\mathbb {S}}}_{ab}| {\mathbb {Z}}\in P({\mathbb {N}}'(a))\cup P({\mathbb {N}}'(b))\}, \forall a \ne b, \end{aligned}$$

where ${\mathbb {N}}'(\cdot )$ is the new neighbor set after deleting the edge $X_i-X_j$. This iterative process deleting edges by satisfying Eq. (6) is followed by updating the recorded separation set based on Eq. (7) until a variable pair presents a significant score decline. Mathematically,

8 $$\begin{aligned} \exists X_a, X_b, rank\left( \max _{{\mathbb {Z}}\in {{\mathbb {T}}}{{\mathbb {S}}}'_{ab}}S(X_a, X_b|{\mathbb {Z}})\right) > k. \end{aligned}$$

Then the *Separation set adjusting* process is applied to find consistent CI hypothesis sets based on the new skeleton. The concept of the wrongly preserved situation is presented in Algorithm 3. As for the wrongly deleted situation, the main idea is to add edges iteratively. The main logic of the wrongly deleted case is shown in Algorithm 4.

#### Early termination

It is possible that for a variable pair $(X_i, X_j)$, no true CI hypothesis can obtain the first *k* highest scores among all tested CI hypotheses. However, just enlarging *k* is inefficient, and thus, an early termination process should be conducted when necessary. The main idea is to record the directions that always conflict with others and make them indirected edges. Specifically, we record the variable pairs that constitute the evidence path to orient its direction for each directed edge. Only variable pairs concerning a v-structure can be recorded. When some conflicts are detected, the most recorded variable pair, i.e., the one that always conflicts with others, lose the ability to form a v-structure. A detailed example is provided in Fig. 8.

**Figure 9:**
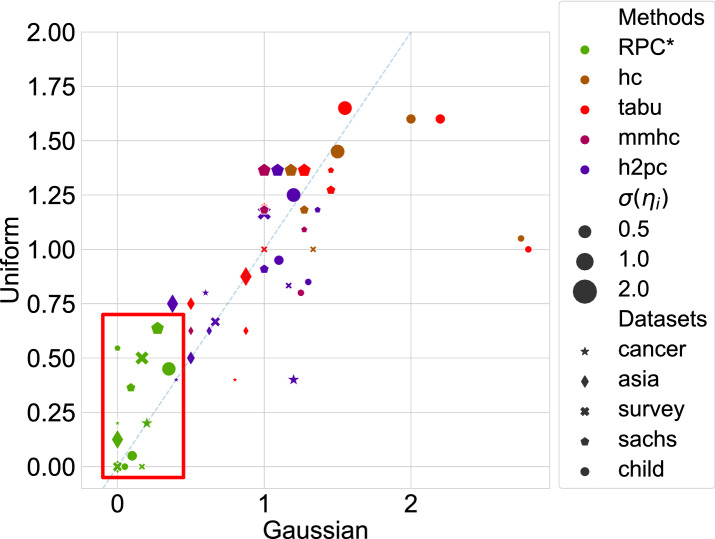
MASHD of DAGs.

Our experiment finds that $T=10$, $\alpha = 0.01$ and $\beta = 10$ can most clearly distinguish the true and the false CI hypotheses. Additionally, the *k* in Eq. (5) is set as 8.

### Evaluation metrics

Consider two graphs defined on the same variable set ${\mathbb {V}}$, $({\mathbb {V}}, {\mathbb {E}})$ and $({\mathbb {V}}, {\mathbb {E}}')$, the SHD of these two graphs is defined as follows:

9 $$\begin{aligned} SHD\left( ({\mathbb {V}}, {\mathbb {E}}), ({\mathbb {V}}, {\mathbb {E}}')\right) {:}{=}|({\mathbb {E}}\cup {\mathbb {E}}')-({\mathbb {E}}'\cap {\mathbb {E}})|+\sum _{e\in {\mathbb {E}}\cap {\mathbb {E}}'}I(d_{\mathbb {E}}(e)\ne d_{{\mathbb {E}}'}(e)). \end{aligned}$$

where $d_{\mathbb {E}}(e)$ denotes the direction of edge *e* in structure ${\mathbb {E}}$ (including the directionless). This means any difference between variable pairs counts for measuring the distance.

Generally, SHD increases with the number of variables (data dimension), i.e., $|{\mathbb {V}}|$. Due to the diversity of the employed datasets, we unify the performance metrics among the various datasets by utilizing the average SHD defined as follows:

10 $$\begin{aligned} \bar{SHD}\left( ({\mathbb {V}}, {\mathbb {E}}), ({\mathbb {V}}, {\mathbb {E}}')\right) {:}{=}\frac{SHD\left( ({\mathbb {V}}, {\mathbb {E}}), ({\mathbb {V}}, {\mathbb {E}}')\right) }{|{\mathbb {V}}|}. \end{aligned}$$

The $\bar{SHD}$ is a scalar change concerning different dataset dimensions and does not discriminate between the algorithms.

Since the direct outputs of RPC* are CPDAGs, i.e., $[{\hat{G}}]$, to straightforwardly compare the algorithms’ performance, we measure the minimal $\bar{SHD}$ between the learned CPDAGs and the CPDAG that the ground truth $G_t$ belonging to, ${\hat{G}}_t$ (termed as minimal $\bar{SHD}$, or MASHD in short). The lower the MASHD, the better the algorithm performs.

11 $$\begin{aligned} \text {MASHD} {:}{=}\min \limits _{{\hat{G}}} \bar{SHD}({\hat{G}}, {\hat{G}}_t). \end{aligned}$$

Unfortunately, MASHD cannot be employed for the second type of dataset since $G_t$ is unknown. For this type of dataset, one popular measurement is KL-divergence^17^. The KL-divergence of a DAG *G* measures the distance between the dataset and the distribution generated by *G*:

12 $$\begin{aligned} KL(G) = \sum _{i=1}^{|{\mathbb {V}}|} H(X_i) -H(\mathbf{X} ) - \sum _{i=1, \mathbf{P} {} \mathbf{a} _i\ne \varnothing }^{|{\mathbb {V}}|} MI(X_i, \mathbf{P} {} \mathbf{a} _i), \end{aligned}$$

where $H(\cdot )$ is the entropy, and $MI(X_i, \mathbf{P} {} \mathbf{a} _i)$ is the mutual information between variable $X_i$ and the parent variables of $X_i$ in *G*, $\mathbf{P} {} \mathbf{a} _i$. Since the entropy does not depend on the DAG, we only compute the mutual information. The higher the mutual information, the better *G* performs. Since the outputs of the CSL algorithms are CPDAGs, i.e., [${\hat{G}}$], we first enumerate all possible orientations of the undirected edges in CPDAGs, i.e., enumerate all $G\in {\hat{G}}$, and select the final output as the one with the maximal average KL-divergence (MAKL-d). The higher the MAKL-d, the better the algorithm performs.

13 $$\begin{aligned} \text {MAKL-d} {:}{=}\max \limits _{G\in {\hat{G}}}\frac{KL(G)}{|{\mathbb {V}}|}. \end{aligned}$$

### Extended performance evaluation

The detailed performance evaluation in Fig. 4a are illustrated in Table 4. Besides, we measure the MASHD between the DAG of the ground truth and the structure learned by algorithms. Since not all algorithms guarantee generating consistent DAGs on all datasets, we only challenge our method against four comparable algorithms. Figure 9 illustrates the performance of all 11 algorithms on all datasets while detailed results are provided in Table 5, with all results rounded to two decimal places and the best performance is in bold type.

**Table 4:** MASHD of CPDAGs.

Structure	Noise	Algorithms										
		RPC*	pc.stable	gs	iamb	fast.iamb	inter. iamb	iamb. fdr	hc	tabu	mmhc	h2pc
Cancer	N(0,0.25)	**0.00**	1.00	1.00	1.00	1.00	1.00	1.00	0.40	0.80	0.40	0.40
	N(0,1)	**0.20**	1.00	0.80	0.80	0.80	0.80	0.80	0.60	1.00	0.60	0.60
	N(0,4)	**0.20**	1.20	1.20	1.20	1.20	1.20	1.00	1.20	1.20	1.20	1.20
	U($-\frac{\sqrt{3}}{2}$, $\frac{\sqrt{3}}{2}$)	**0.20**	1.00	1.00	1.00	1.00	1.00	1.00	0.40	0.40	0.40	0.40
	U($-\sqrt{3}$, $\sqrt{3}$)	**0.20**	1.00	1.20	1.20	1.20	1.20	0.80	0.80	1.20	0.80	0.80
	U($-2\sqrt{3}$, 2$\sqrt{3}$)	**0.20**	1.00	1.00	1.00	1.00	1.00	1.00	0.40	0.40	0.40	0.40
Survey	N(0,0.25)	**0.83**	1.17	1.50	1.50	1.50	1.50	1.33	1.33	1.00	1.17	1.17
	N(0,1)	**0.00**	1.67	1.67	1.67	1.67	1.67	1.67	0.67	0.67	0.67	0.67
	N(0,4)	**0.17**	1.33	1.33	1.33	1.33	1.33	1.17	1.00	1.00	1.00	1.00
	U($-\frac{\sqrt{3}}{2}$, $\frac{\sqrt{3}}{2}$)	**0.00**	1.17	1.17	1.17	1.17	1.17	1.17	1.00	1.00	0.83	0.83
	U($-\sqrt{3}$, $\sqrt{3}$)	**0.00**	1.00	1.17	1.17	1.17	1.17	1.17	0.67	0.67	0.67	0.67
	U($-2\sqrt{3}$, 2$\sqrt{3}$)	**0.83**	1.67	1.67	1.67	1.67	1.67	1.67	1.67	1.67	1.67	1.67
Asia	N(0,0.25)	**0.00**	1.13	0.88	0.88	0.88	0.88	0.75	1.00	1.00	0.63	0.70
	N(0,1)	**0.00**	0.63	0.38	0.38	0.38	0.38	0.50	0.63	0.63	0.63	0.63
	N(0,4)	**0.00**	0.75	0.88	0.88	0.88	0.88	1.00	0.50	0.88	0.50	0.50
	U($-\frac{\sqrt{3}}{2}$, $\frac{\sqrt{3}}{2}$)	**0.00**	1.13	0.75	0.75	0.75	0.75	0.75	0.75	0.75	0.75	0.75
	U($-\sqrt{3}$, $\sqrt{3}$)	**0.00**	0.75	0.88	0.88	0.88	0.88	0.88	0.63	0.75	0.63	0.63
	U($-2\sqrt{3}$, 2$\sqrt{3}$)	**0.38**	0.75	1.13	1.13	1.13	1.13	1.13	0.88	1.00	0.88	0.88
Sachs	N(0,0.25)	**0.00**	1.82	1.91	1.91	1.91	1.91	2.00	2.45	2.45	2.00	2.00
	N(0,1)	**0.09**	1.82	1.64	1.64	1.64	1.64	1.64	2.18	2.00	1.73	1.73
	N(0,4)	**0.91**	1.82	1.73	1.73	1.73	1.73	1.73	2.00	1.91	1.82	1.91
	U($-\frac{\sqrt{3}}{2}$, $\frac{\sqrt{3}}{2}$)	**1.00**	1.91	2.00	2.00	2.00	2.00	1.82	2.18	2.18	1.91	2.00
	U($-\sqrt{3}$, $\sqrt{3}$)	**0.82**	1.82	1.91	1.91	2.00	1.91	1.91	2.00	2.00	1.91	1.91
	U($-2\sqrt{3}$, 2$\sqrt{3}$)	**1.18**	1.73	1.64	1.64	1.64	1.64	1.64	1.91	1.91	1.91	1.91
Child	N(0,0.25)	**0.15**	1.50	2.00	1.90	2.00	1.90	1.95	3.15	3.15	1.55	1.60
	N(0,1)	**0.55**	1.70	1.65	1.65	1.70	1.65	1.70	2.45	2.50	1.55	1.55
	N(0,4)	**0.60**	1.60	1.45	1.45	1.65	1.45	1.70	1.60	1.65	1.45	1.45
	U($-\frac{\sqrt{3}}{2}$, $\frac{\sqrt{3}}{2}$)	**0.00**	1.20	0.90	0.90	0.90	0.90	1.05	1.45	1.40	1.25	1.20
	U($-\sqrt{3}$, $\sqrt{3}$)	**0.50**	1.60	1.50	1.55	1.55	1.55	1.35	2.05	2.05	1.45	1.45
	U($-2\sqrt{3}$, 2$\sqrt{3}$)	**0.60**	1.65	1.55	1.55	1.55	1.55	1.60	1.75	1.90	1.55	1.55

Significant values are in bold.

**Table 5:** MASHD of DAGs and MAKL-d.

Structure	Noise	Algorithms				
		RPC*	hc	tabu	mmhc	h2pc
cancer	N(0,0.25)	**0.00**/**1.05**	0.40/0.90	0.80/0.90	0.40/0.90	0.40/0.90
	N(0,1)	**0.20**/0.56	0.60/**0.59**	1.00/**0.59**	0.60/**0.59**	0.60/**0.59**
	N(0,4)	**0.20**/0.75	1.20/**0.80**	1.20/**0.80**	1.20/**0.80**	1.20/**0.80**
	U($-\frac{\sqrt{3}}{2}$, $\frac{\sqrt{3}}{2}$)	**0.20**/0.67	0.40/**0.92**	0.40/**0.92**	0.40/**0.92**	0.40/**0.92**
	U($-\sqrt{3}$, $\sqrt{3}$)	**0.20**/0.66	0.80/**1.08**	1.20/0.92	0.80/**1.08**	0.80/**1.08**
	U($-2\sqrt{3}$, 2$\sqrt{3}$)	**0.20**/0.67	0.40/**1.00**	0.40/**1.00**	0.40/**1.00**	0.40/**1.00**
survey	N(0,0.25)	**0.17**/**1.21**	1.33/1.10	1.00/1.00	1.17/0.91	1.17/0.91
	N(0,1)	**0.00**/**0.90**	0.67/0.61	0.67/0.60	0.67/0.61	0.67/0.61
	N(0,4)	**0.17**/**0.64**	1.00/0.30	1.00/0.30	1.00/0.30	1.00/0.30
	U($-\frac{\sqrt{3}}{2}$, $\frac{\sqrt{3}}{2}$)	**0.00**/**0.95**	1.00/0.74	1.00/0.77	0.83/0.55	0.83/0.55
	U($-\sqrt{3}$, $\sqrt{3}$)	**0.00**/**1.02**	0.67/0.72	0.67/0.72	0.67/0.72	0.67/0.72
	U($-2\sqrt{3}$, 2$\sqrt{3}$)	**0.50**/**0.83**	1.17/0.23	1.17/0.23	1.17/0.23	1.17/0.23
asia	N(0,0.25)	**0.00**/**1.07**	0.88/0.87	0.88/0.87	0.50/0.89	0.63/0.88
	N(0,1)	**0.00**/0.87	0.50/**0.92**	0.50/**0.92**	0.50/**0.92**	0.50/**0.92**
	N(0,4)	**0.00**/**0.93**	0.38/0.77	0.88/0.74	0.38/0.77	0.38/0.77
	U($-\frac{\sqrt{3}}{2}$, $\frac{\sqrt{3}}{2}$)	**0.00**/**1.15**	0.63/1.00	0.63/1.00	0.63/1.00	0.63/1.00
	U($-\sqrt{3}$, $\sqrt{3}$)	**0.00**/**1.11**	0.50/0.92	0.75/0.96	0.50/0.92	0.50/0.92
	U($-2\sqrt{3}$, 2$\sqrt{3}$)	**0.13**/**1.25**	0.75/0.90	0.88/0.96	0.75/0.90	0.75/0.90
sachs	N(0,0.25)	**0.00**/**1.51**	1.45/1.13	1.45/1.13	1.23/1.00	1.36/0.93
	N(0,1)	**0.09**/**1.27**	1.27/1.01	1.45/0.84	1.00/0.77	1.00/0.79
	N(0,4)	**0.27**/**1.20**	1.18/0.98	1.27/0.97	1.00/0.84	1.09/0.91
	U($-\frac{\sqrt{3}}{2}$, $\frac{\sqrt{3}}{2}$)	**0.55**/**1.11**	1.27/1.06	1.36/1.06	1.09/1.00	1.18/1.07
	U($-\sqrt{3}$, $\sqrt{3}$)	**0.36**/**1.31**	1.18/1.06	1.27/1.06	1.18/1.07	0.91/1.12
	U($-2\sqrt{3}$, 2$\sqrt{3}$)	**0.64**/**1.29**	1.36/0.82	1.36/0.82	1.36/0.87	1.36/0.87
child	N(0,0.25)	**0.05**/1.11	2.75/**1.42**	2.80/1.36	1.25/1.16	1.30/1.21
	N(0,1)	**0.10**/1.05	2.00/**1.08**	2.20/1.07	1.10/**1.08**	1.10/1.07
	N(0,4)	**0.35**/**0.91**	1.50/0.83	1.55/0.83	1.20/0.87	1.20/0.87
	U($-\frac{\sqrt{3}}{2}$, $\frac{\sqrt{3}}{2}$)	**0.00**/1.13	1.05/**1.19**	1.00/**1.19**	0.80/1.14	0.85/1.10
	U($-\sqrt{3}$, $\sqrt{3}$)	**0.05**/**1.17**	1.60/1.13	1.60/1.13	0.95/1.13	0.95/1.08
	U($-2\sqrt{3}$, 2$\sqrt{3}$)	**0.45**/**1.14**	1.45/1.04	1.65/1.09	1.25/0.96	1.25/0.96

Significant values are in bold.

The numbers before (after) forward slashes are MASHD (MAKL-d).
